# Inflammatory burden index as a prognostic marker in patients with advanced gastric cancer treated with neoadjuvant chemotherapy and immunotherapy

**DOI:** 10.3389/fimmu.2024.1471399

**Published:** 2025-01-21

**Authors:** Jiao-Bao Huang, Zhi-Yong Zhou, Jun Lu, Ji-Yun Zhu, Bin Lai, Sheng-Xun Mao, Jia-Qing Cao

**Affiliations:** ^1^ Department of Gastrointestinal Surgery, The Second Affiliated Hospital of Nanchang University, Nanchang, China; ^2^ Department of Gastric Surgery, Fujian Medical University Union Hospital, Fuzhou, China; ^3^ Department of Gastrointestinal Surgery, The First Affiliated Hospital of Ningbo University, Ningbo, China

**Keywords:** gastric cancer, neoadjuvant therapy, inflammatory index, immunotherapy, prognosis

## Abstract

**Background:**

Blood inflammation index has been shown to correlate with the prognosis of patients with gastric cancer. However, few studies have compared the efficacy of existing blood inflammatory markers in predicting the prognosis of patients with locally advanced gastric cancer in combination with neoadjuvant chemotherapy and immunotherapy.

**Objective:**

The objective of this study was to compare the prognostic value of existing commonly used blood inflammatory index in patients with advanced gastric cancer treated with neoadjuvant chemotherapy combined with immunotherapy.

**Methods:**

The clinicopathological data of patients with advanced gastric cancer from three centers in China were analyzed retrospectively. Univariate COX regression analysis was used to analyze the independent risk factors of poor tumor regression and overall survival (OS) in this part of patients, and the predictive value of different inflammatory indexes on prognosis was compared by C-index index. Finally, Inflammatory burden index(IBI) was grouped by X-tile software, and Kaplan-Meier method was used to compare the survival difference between groups.

**Results:**

A total of 163 patients were enrolled in this study. The median age was 63 years(56-68). The median cycle of neoadjuvant therapy was 4(3-4). The median survival time was 85.1%(1 years), 65.6%(2 years), and 47.4%(3 years).Univariate analysis showed that IBI was an independent risk factor for non-TR(residual tumor cells>50%) (HR=1.08,95%CI:1.00-1.45,p<0.001)and OS(HR=1.04,95%CI:1.03-1.05,p<0.001). IBI is the best predictor of OS (C-index: 0.82, 95% CI: 0.78-0.87) among all inflammatory indexes. The IBI cutoff value was 52.1. It was found that the high IBI group had a higher incidence of postoperative complications(32.1%vs14.3%, p=0.001), the proportion of non-TR patients was significantly higher than that of the low IBI group(64.3%vs35.7%, p =0.001), and the high IBI group had a significantly lower OS((47.6% vs 87.6%, p < 0.001).

**Conclusion:**

IBI is the best inflammatory index to predict the prognosis of advanced gastric cancer treated with neoadjuvant chemotherapy combined with immunotherapy, which will help guide patients’ treatment decisions. This result still needs to be verified by large prospective studies.

## Introduction

Gastric cancer is the fifth and fourth malignant tumor in the world in terms of morbidity and mortality (1). In China, the mortality rate is higher (ranking third) (2), because the tumor was in the middle and late stage at the time of diagnosis. For patients with advanced gastric cancer, the 5-year survival rate of patients with stage II is 61-63%, while that of patients with stage III is only 30-35%, even with surgical resection and postoperative adjuvant radiotherapy and chemotherapy (3). The importance of comprehensive treatment for patients with advanced gastric cancer has been paid more and more attention by scholars at home and abroad.

Immunocheckpoint inhibitors (ICIs) are more and more widely used in gastric cancer. The research results of CheckMate-649 and ATTRACTION-02 show that nivolumab has achieved good survival benefits in the treatment of gastric cancer (4, 5). At present, the application of ICIs combined with chemotherapy regimen in neoadjuvant therapy for patients with locally advanced gastric cancer has been carried out in many centers. In two studies reported in 2021 (NCT04341857 and NCT04065282), sindilizumab combined with FLOT (fluorouracil + oxaliplatin + docetaxel + leucovorin) and XELOX (oxaliplatin + capecitabine) were used as neoadjuvant therapy for gastric cancer, with postoperative pCR rates of 18.8% and 23.1%, respectively. MPR rates were 62.5% and 53.8%, and the treatment was well tolerated (6, 7). However, from the current research, not all patients can benefit from ICIs. CPS≥5 and dMMR/MSI-H are effective biomarkers for predicting the efficacy of ICIs in patients with gastric cancer (8), but their expression rate is only 8-15% (9), and their detection cost and inconvenience also limit their application. It is urgent to explore a biomarker that can predict the efficacy of ICIs simply and quickly.

If a malignant tumor is a wound that never heals, then the most representative interaction between tumor and host is systemic inflammatory reaction (10). Systemic inflammation is an important feature of tumor microenvironment, which plays a vital role in the disease progression and prognosis of tumor patients (11).

Hematological inflammatory indexes, such as neutrophils, lymphocytes, platelets and C-reactive protein (CRP), can effectively reflect the systemic inflammatory state of tumors (12, 13). Many studies have evaluated several biomarkers of systemic inflammation composed of these inflammatory indexes, and proved that these biomarkers have important prognostic value in different cancers, including gastric cancer (14–16). As a new inflammatory index, Inflammatory burden index(IBI) is increasingly recognized by clinicians. For example, in patients with gastric cancer undergoing surgical treatment, the 5-year OS and DFS of patients with elevated IBI at diagnosis were significantly lower than those of patients with low IBI(OS: 79.07%vs 70.00%,p < 0.0048;DFS: 74.42%% vs 50.00%,p < 0.0082). Therefore, IBI is a promising new biomarker for gastric cancer. However, the prognostic value of IBI in the treatment of new therapies such as neoadjuvant immunization combined with chemotherapy is still unclear. Therefore, the purpose of this study is to compare the prognostic value of IBI with existing systemic inflammatory biomarkers through multi-center retrospective analysis, and to determine the best systemic inflammatory biomarker for patients with locally advanced gastric cancer.

## Method

### Study population

**Figure 1:**
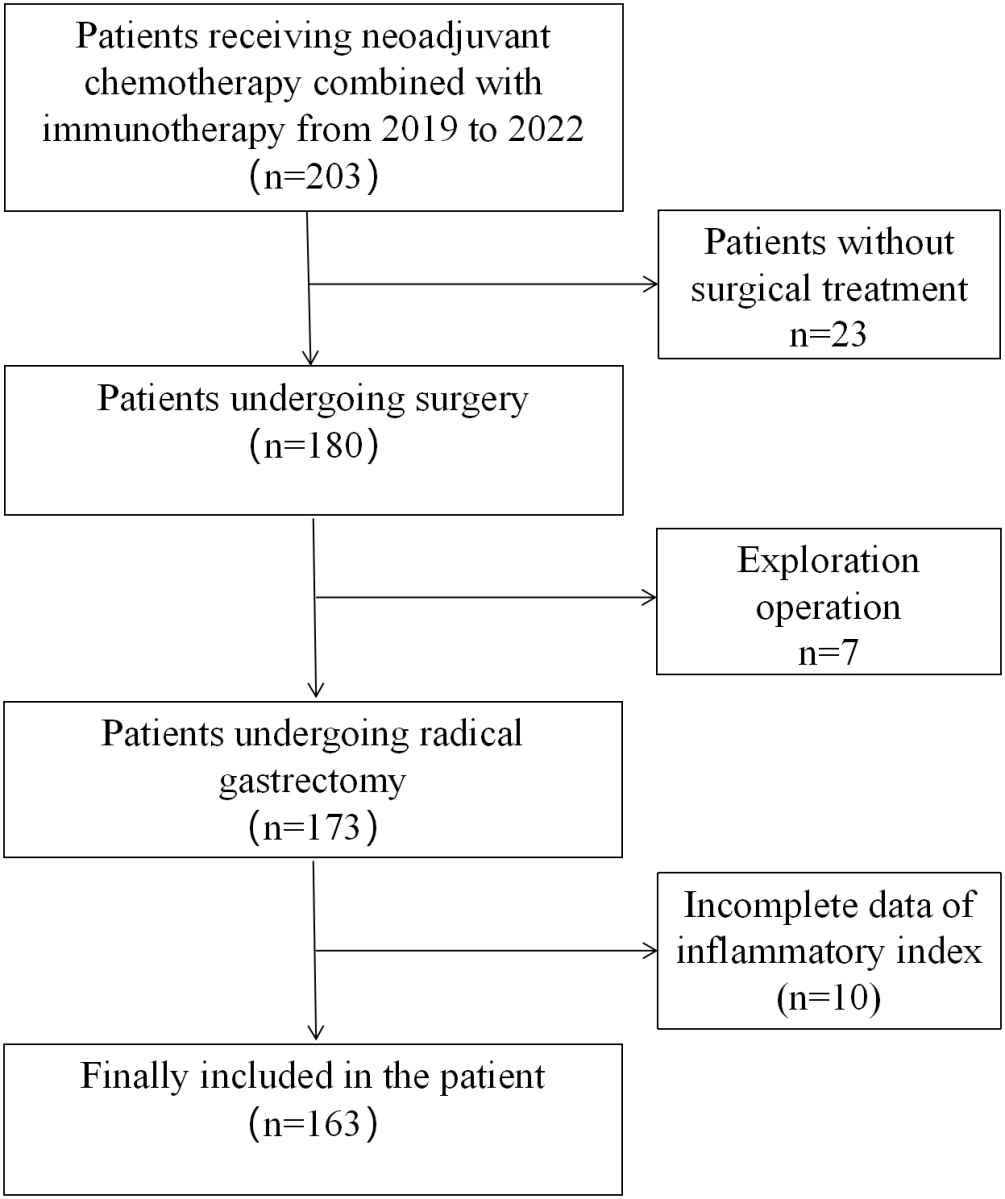
Study flow chart.

A total of 203 patients with locally advanced gastric cancer who received neoadjuvant chemotherapy combined with immunotherapy from October 2019 to December 2022 from three large medical teaching hospitals in China were enrolled in this study. Patients meeting the following criteria were included in the study:(1) aged 18-75 years; (2) Histopathologically confirmed primary gastric adenocarcinoma, clinical stage: cT2-4, lymph node N0-N3, no distant metastasis (M0); (3) have not received chemotherapy (radiotherapy) or other anti-tumor therapy within 6 months; (4) At the time of diagnosis, there were relevant inflammation-related indexes; Those who meet the following criteria are excluded: (1) malignant diseases combined with other organs; (2) Evidence of peritoneal spread or distant metastasis (including intraoperative exploration findings after neoadjuvant therapy); (3) Previous gastrectomy or endoscopic submucosal dissection (ESD). (4) Patients who have not received surgery; (5) Patients with incomplete clinical and pathological data; A total of 30 patients without surgery were excluded (including 7 patients with abdominal implant metastasis found in surgical exploration), and the inflammatory index data of 10 patients were incomplete. Finally, a total of 163 patients were included in the analysis (**Figure 1**). The study was reviewed and approved by the Ethics Committee of the Second Affiliated Hospital of Nanchang University(Ethic pre-approval number: I-Medical Research and Ethics Review [2024] No. (60)).

### Data collection

Collect the patient’s baseline data including gender, age, body mass index, complications, Eastern Cooperative Oncology Group (ECOG), operation type, neoadjuvant therapy cycle and grade of tumor regression (TRG hierarchical), tumor TNM staging. Fasting venous blood was collected from the patients in their respective central laboratories within 1 week before receiving anti-cancer treatment and serological index was tested. Haematological indices include leukocytes, neutrophils, lymphocytes, monocytes, C-reactive protein, platelets, hemoglobin, and albumin. Inflammatory burden index (IBI) = C-reactive protein (mg/L)*neutrophil (uL)/lymphocyte (uL). The others calculation methods of inflammatory indexes in the study are shown in **Supplementary Table 1**.

### Neoadjuvant chemotherapy combined with immunotherapy

The neoadjuvant treatment regimen is fluorouracil based chemotherapy combined with ICIs, and the chemotherapy regimen is generally SOX/XELOX regimen of 2-4 cycles (17) (S-140-60mg or capecitabine 1000mg/m^2^, orally, twice a day, on day 1-14, oxaliplatin 130mg/m^2^ intravenous injection on day 1). FOLFOX4 regimen of 2-4 cycles (18) (Day 1: Oxaliplatin 85mg/m^2^, calcium folinate 200mg/m^2^ for 2 hours, fluorouracil 400mg/m^2^ for 22 hours and fluorouracil 600mg/m^2^ for 22 hours. Immunosuppressants were administered on the first day of chemotherapy along with the chemotherapy cycle (the drug dose was determined according to the patient’s body surface area(Body surface area (m2)= 0.0061× height (cm)+0.0128× weight (kg)-0.1529) (19). For patients with severe hematotoxicity or non-hematotoxicity, the dose will be appropriately reduced). The next cycle of chemotherapy was repeated on the 22nd day. Surgery was performed at least 3 weeks after the completion of neoadjuvant therapy. All surgical procedures, including the extent of lymph node dissection, were performed according to the guidelines of the Japanese Society for Gastric Cancer Research (20), while staging was performed according to TNM classification (AJCC, 8th edition) (21). The posttreatment pathologic response was quantified using Becker regression criteria, which is based on an estimate of the percentage of live tumor cells relative to the visually identifiable tumor bed. The following categories are included:TRG1a(no residual tumor cells), TRG1b(< 10% residual tumor cells), TRG2 (10-50% residual tumor cells), and TRG3 (> 50% residual tumor cells) (22). In this study, TRG grade 1a/1b/2 was considered tumor regression(TR), and the TRG3 grade was considered non-tumor regression(non-TR).

### Surgical quality control and follow-up evaluation

All patients were treated by experienced surgeons who had passed the learning curve and completed more than 50 laparoscopic radical procedures for gastric cancer (23). The surgical procedures followed the Japanese guidelines for gastric cancer treatment, including laparoscopic radical gastrectomy and D2 lymph node dissection (20).

Each patient was assessed at a follow-up of at least 36 months. Follow-up was conducted every 3 months for the first 2 years and every 6 months thereafter. Most routine follow-up appointments include a physical exam, laboratory tests (including CA199, CA72-4, and CEA measurements), chest radiography, abdominal and pelvic ultrasound or computed tomography, and annual endoscopy. Overall survival was defined as death from any cause from diagnosis (24).

### Statistical analysis

The primary endpoint was overall survival. The secondary endpoint was postoperative complications of tumor regression grade. The data are described as the absolute number and percentage of normally distributed variables, as the mean and standard deviation (SD), or as the median and interquartile range (IQR). The classified variables were tested by the chisquare test or Fisher’s exact test, and the continuous variables were compared by t test. COX regression model was used for univariate and multivariate analysis. C-index and time-dependent ROC curve were used to compare the effects of inflammatory indexes on prognosis. Using X-tile to obtain the best cutoff value of IBI (X-tile was developed by Yale University as a bioinformatics tool for biomarker evaluation and result optimization the working principle is to distinguish the final population subsets and the associated Kaplan-Meier curve through the log-rank test) (25), Cox proportional risk regression model for evaluating the hazard ratio of various risk factors. A p value lower than 0.05 was considered statistically significant. For statistical analysis, SPSS software (version 22.0, Stanford, CA, USA) and R version 3.6.0 (R Foundation for Statistical Computing, Auckland, New Zealand) were used.

## Result

### General clinical and pathological data of patients

A total of 163 patients were included in the Second Affiliated Hospital of Nanchang University, the Union Medical College Hospital of Fujian Medical University and the First Affiliated Hospital of Ningbo University who received neoadjuvant chemotherapy combined with immunotherapy and underwent gastrectomy from October 2019 to December 2022.There were 115 males (70.6%) and 48 females (29.4%), with a median age of 63 years (56-68), a median of 4 cycles of neoadjuvant therapy (3-4 cycles), non-TR=68 cases (41.7%), 29 cases (17.8%) of postoperative complications, and 106 cases (65%) of treatment-related adverse reactions. The median IBI value was 19.9(6.3, 63.5). **Table 1** summarizes other inflammatory indexes and the clinicopathological information of all patients.

**Table 1:** General characteristics of patients.

Characteristic	Total = 163^1^
Gender, (%)	
Female	48 (29.4%)
Male	115 (70.6%)
Age, year	63.0 (56.0, 68.0)
BMI, Kg/m2	23.3( ± 2.9)
PD1 cycle	4 (3, 4)
ypT, (%)	
1	16 (9.8%)
2	26 (16.0%)
3	74 (45.4%)
4	47 (28.8%)
ypN, (%)	
0	29 (17.8%)
1	53 (32.5%)
2	35 (21.5%)
3	46 (28.2%)
TRG, (%)	
0	18 (11.2%)
1	30 (18.3%)
2	47 (28.8%)
3	68 (41.7%)
Surgery method, (%)	
Distal gastrectomy	56 (34.4%)
Total gastrectomy	107 (65.6%)
Postoperative complication, No. (%)	
Yes	29(17.8%)
>No	134(82.2%)
Treatment-related adverse events, No. (%)	
Yes	106(65.0%)
No	57(35.0%)
CRP	7.3 (2.8, 14.4)
IBI	19.9 (6.3, 63.5)
mGPS, (%)	
0	121 (74.2%)
1	15 (9.2%)
2	27 (16.6%)
NLR	2.9 (1.9, 4.9)
NPR	2.0 (1.3, 2.6)
SII	372 (216, 868)
PLR	109.8 (82.1, 190.7)

^1^n (%); Median (IQR); Mean( ± SD).

### Univariate and multivariate COX regression analysis of the influencing factors of inflammatory index on prognosis

Univariate analysis found that clinical stage III, CRP, IBI and SII were the influencing factors of non-TR, while multivariate analysis found that only IBI (HR = 1.08, 95% CI: 1.00-1.45, p < 0.001) and cTNM (HR=1.76,95%CI:1.65-3.36, p=0.027) (**Table 2**).Univariate COX analysis showed that ypTNM staging, CRP, IBI and PLR were the influencing factors of OS, while multivariate analysis showed that only IBI (HR = 1.04, 95% CI: 1.03-1.05, p < 0.001) and ypTNM (HR=1.86,95%CI:1.25-3.49, p=0.036) were involved (**Table 3**).

**Table 2:** Univariate and multivariable analysis of the relationship between inflammatory indexes with non-TR.

Characteristic	Univariate			Multivariate		
	HR^1^	95% CI^1^	P.value	HR^1^	95% CI^1^	P.value
Gender			0.195			
Female	Ref.	Ref.				
>Male	0.67	0.38, 1.21				
Age	0.98	0.96, 1.01	0.170			
BMI	0.93	0.83, 1.05	0.243			
ECOG						
0	Ref.	Ref.	0.225			
≥1	0.96	0.88,1.45				
cTNM			**0.047**			**0.027**
Stage II	Ref.	Ref.		Ref.	Ref.	
Stage III	1.56	1.23, 2.55		1.76	1.65, 3.36	
Tumor differentiation						
Low/Middle	Ref.	Ref.				
High	1.35	0.91, 2.87				
CRP	1.04	1.02, 1.05	**<0.001**	1.00	0.98,1.02	0.78
IBI	1.12	1.01, 1.18	**<0.001**	1.08	1.00,1.45	**<0.001**
mGPS			0.445			
0	Ref.	Ref.				
1	1.27	0.50, 3.27				
2	1.63	0.78, 3.41				
NLR	1.02	0.92, 1.14	0.684			
NPR	1.16	0.92, 1.45	0.234			
SII	1.00	1.00, 1.00	**0.009**	1.00	1.00,1.00	0.53
PLR	1.01	1.01, 1.03	0.113			

^1^HR, Hazard Ratio; CI, Confidence Interval. The bold values ​​indicate statistical significance.

**Table 3:** Univariate and multivariable analysis of the relationship between inflammatory indexes with OS.

Variable		Univariate			Multivariate	
	HR^1^	95% CI^1^	P.value	HR^1^	95% CI^1^	P.value
Gender			0.983			
Female	Ref.	Ref.				
Male	1.01	0.55, 1.83				
Age	1.00	0.97, 1.02	0.832			
BMI	0.98	0.86, 1.11	0.762			
ECOG						
0	Ref.	Ref.	0.315			
≥1	1.06	0.78,2.35				
ypTNM			**0.023**			**0.036**
Stage I	Ref.	Ref.		Ref.	Ref.	
Stage II	0.51	0.23, 1.14		0.81	0.43, 1.36	
Stage III	1.68	1.04, 2.36		1.86	1.25, 3.49	
Surgery method			0.213	0.83	0.42, 1.63	0.59
Distal gastrectomy	Ref.	Ref.				
Total gastrectomy	0.47	0.26, 1.24				
TRG			0.312			
0	Ref.	Ref.				
1	1.41	0.51, 3.89				
2	2.23	0.89, 5.64				
3	1.49	0.57, 3.88				
CRP	1.04	1.03, 1.06	**<0.001**	1.00	0.98, 1.02	0.99
NEU	0.93	0.81, 1.08	0.302			
PLT	1.00	1.00, 1.00	0.256			
ALB	0.99	0.94, 1.04	0.602			
IBI	1.04	1.03, 1.05	**<0.001**	1.04	1.03, 1.05	**<0.001**
mGPS			0.356			
0	Ref.	Ref.				
1	0.98	0.35, 2.78				
2	1.72	0.84, 3.49				
NLR	1.08	0.99, 1.19	0.118			
NPR	1.23	0.87, 1.42	0.121			
SII	1.08	0.95, 1.00	<0.096			
PLR	0.99	0.99, 1.00	**<0.001**	1.00	0.99, 1.00	0.36

^1^HR, Hazard Ratio; CI, Confidence Interval. The bold values ​​indicate statistical significance.

### Comparison of the effects of different inflammatory indices on prognosis

**Figure 2:**
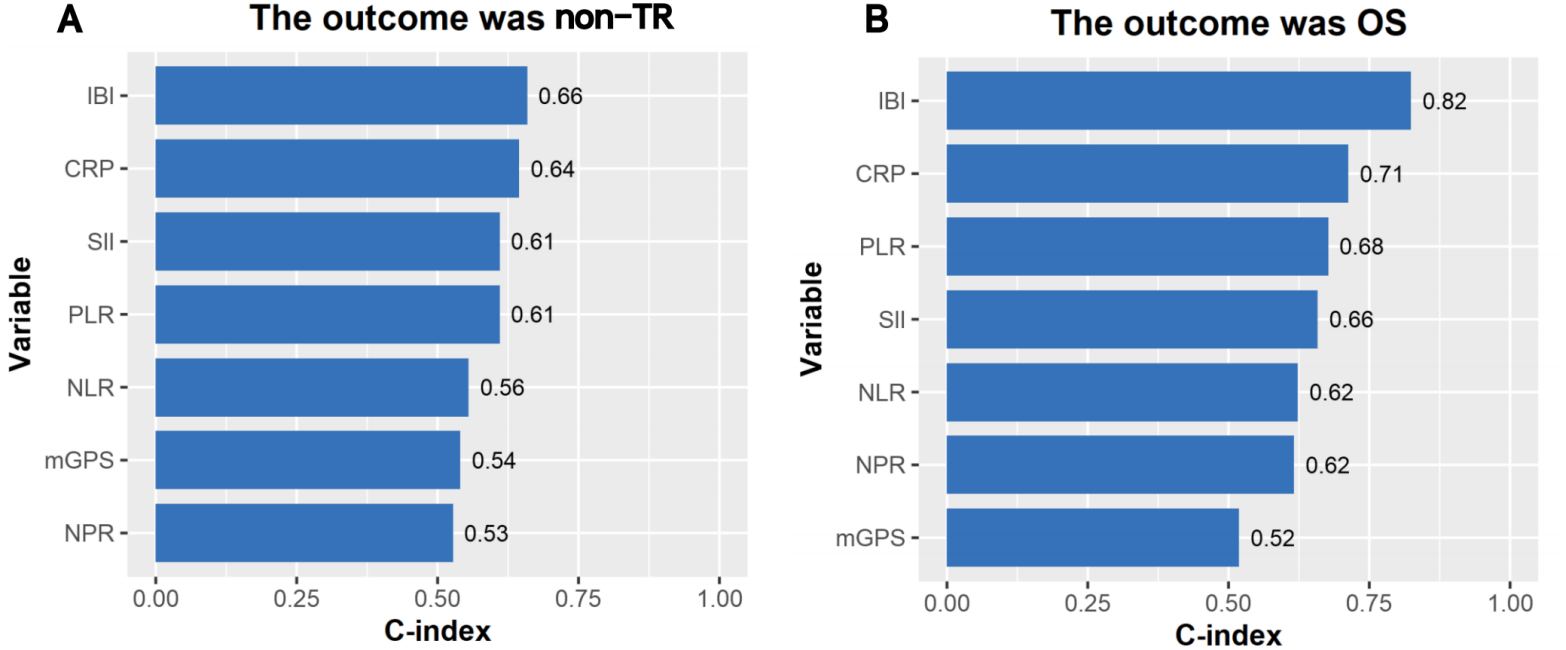
**(A, B)** Consistency test on the accuracy of inflammatory indexes in prognosis.

**Figure 3:**
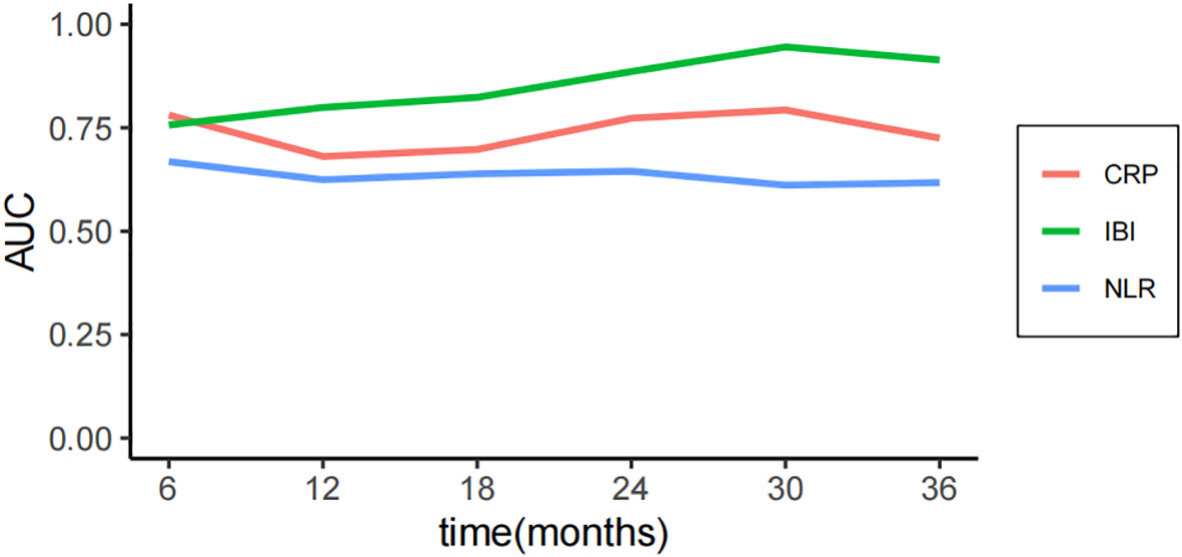
Time-dependent ROC curves comparing the predictive performance for OS.

The median follow-up time was 30 months, and the OS of 1, 2 and 3 years was 85.1%, 65.6% and 47.4% respectively. The consistency test analysis shows that IBI > CRP > SII = PLR > NLR > MGPS > NPR (C-index value: IBI=0.66, CRP=0.64, SII=0.61, PLR=0.61, NLR=0.56, mGPS=0.54, NPR=0.53).In OS, IBI > CRP > PLR > SII > NLR > NPR > mGPS (C-index: IBI=0.82, CRP=0.71, PLR=0.68, SII=0.66, NLR=0.62, NPR=0.62, mGPS=0.52) (**Figure 2**). The time-dependent ROC curve compared the inflammation index of each group in predicting OS, and it was also found that IBI was superior to other indexes (**Figure 3**; **Supplementary Figure 1**).

### Comparison of prognostic differences between high and low IBI groups

**Figure 4:**
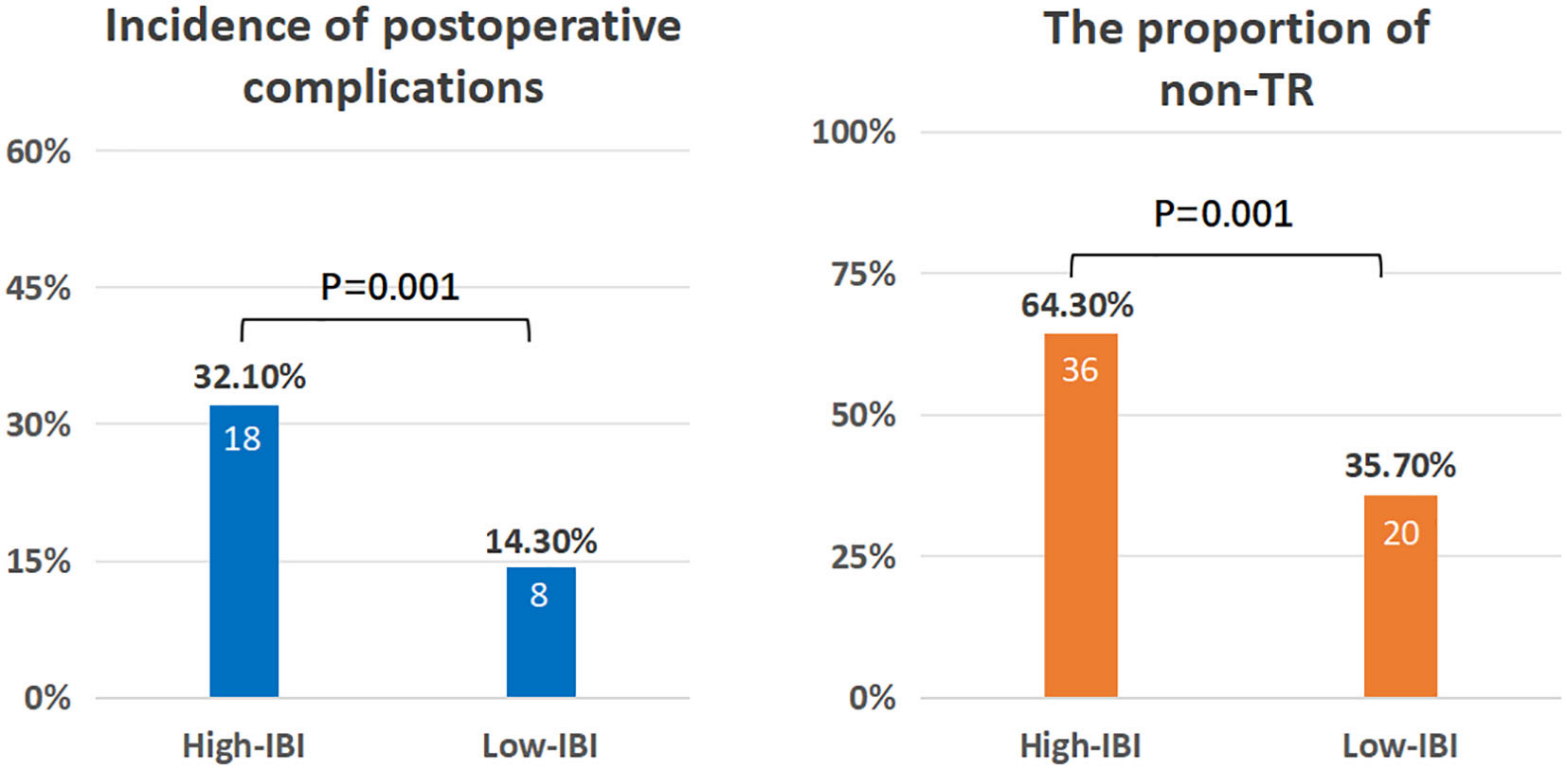
Comparison of short-term prognosis between the two groups.

**Figure 5:**
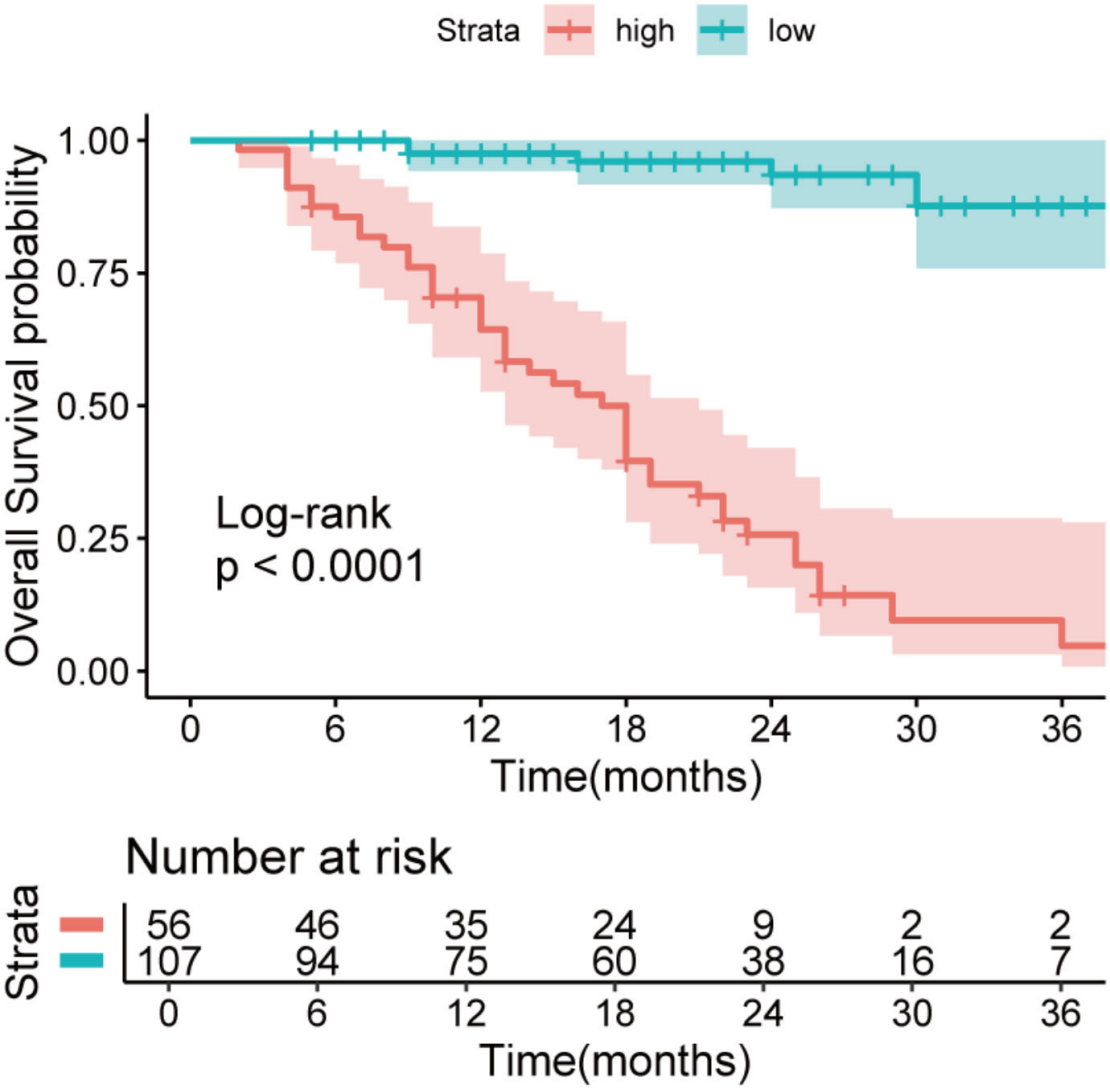
Kaplan-Meier Survival curve to visualize the survival difference between the two groups.

The cutoff value of IBI obtained by X-tile software was 52.1 (**Supplementary Figure 2**), among which 56 cases (34.4%) in the high IBI group and 107 cases (65.6%) in the low IBI group.(There were 13 censored cases in the H-IBI group, accounting for approximately 24.1%, and 32 censored cases in the L-IBI group, accounting for approximately 30.2%).After balancing the baseline data of the two groups by 1:1 propensity matching (**Supplementary Tables 2**, **3**), the incidence of postoperative complications and the proportion of non-TR patients in the high IBI group were significantly higher than those in the low IBI group (Postoperative complications: 32.1%vs14.3%, p=0.001, non-TR: 64.3%vs35.7%, p = 0.001) (**Figure 4**).Kaplan-Meier curve Intention-to-treat analysis found that the 3-year survival rate in the overall population was significantly lower than that in the low-IBI group (47.6% vs 87.6%, p < 0.001) (**Figure 5**).

## Discussion

In this multicentered, retrospective analysis, we compared the effect of existing inflammatory indices on neoadjuvant immunotherapy in patients with locally advanced gastric cancer, and found that IBI was most closely associated with prognosis, and patients with high IBI were significantly worse than those with low IBI in both short-term and long-term outcomes. The results will be conducive to the preliminary judgment of the therapeutic effect of neoadjuvant therapy for gastric cancer patients, and can guide the clinical treatment decision of locally advanced gastric cancer patients.

In recent years, with the development of immunotherapy, treatment options for cancer have changed dramatically. A key challenge, however, is selecting patients who are most likely to respond. Existing studies have shown that PD-L1 expression, MSI status, and tumor mutation load are related to immunotherapy response (26–28), but these tests are cumbersome and expensive. Inflammation index is a commonly used method to evaluate the inflammatory status of patients in clinical practice, which has the characteristics of convenience and low cost. There have been a number of previous studies on the evaluation of inflammation index and immunotherapy efficacy. For example, Riedl et al. found that the efficacy of PD-L1 in patients with advanced non-small cell lung cancer was negatively correlated with the level of CRP before treatment (29). Sui et al. found that in patients with colorectal cancer, the higher the NLR, the worse the efficacy of ICIs (30).

The IBI is a new inflammatory parameter that has recently been used to assess negative inflammatory syndrome in cancer patients. In a study comparing more than 6000 cancer patients, Xie et al. found that the survival of patients with high IBI was significantly worse than that of patients with low IBI (45.7%vs69.1%; P<0.001) (31). In specific cancers, Shi et al. found that lung cancer patients with high IBI had significantly worse survival after surgical resection than patients with low IBI (35.46%vs.57.22%; P<0.001) (32). Zhao et al. also obtained the same result in gastric cancer (33). However, there are few reports of neoadjuvant immunotherapy for IBI in patients with locally advanced gastric cancer. According to our results, IBI predicted the prognosis of patients with neoadjuvant immunotherapy better than other inflammatory indices, and the high IBI group had worse tumor regression, higher incidence of postoperative complications and worse overall survival than the low IBI group. This suggests that the inflammatory burden index may be related to the efficacy of immunotherapy.

The occurrence of tumor not only depends on the individual characteristics of the tumor, but also depends on the host’s systemic immune inflammatory response (34). There is increasing evidence that blood-derived systemic inflammatory biomarkers are effective predictors of prognosis of various cancers (35, 36). Serum CRP is the most representative clinical marker of acute systemic inflammation (37). Neutrophils secrete inflammatory mediators and chemokines, creating a tumor microenvironment suitable for tumor proliferation, invasion and microvascularization, and promoting tumor occurrence and development (38, 39). Lymphocytes play an important role in cancer immune monitoring, inhibiting tumor cell proliferation and growth through cytokine mediated cytotoxicity (40). Compared with CRP and NLR alone, IBI represents the balance between acute inflammation and immune inflammation. Compared with other markers, although they all have inflammation-related indicators, their combination fails to reflect immune-related characteristics, such as SII, PLR and NPR, while mGPS pays more attention to the nutritional status of patients. Therefore, we believe that IBI combined with CRP, neutrophils and lymphocytes can better predict the efficacy of neoadjuvant immunotherapy for advanced gastric cancer than other inflammatory indices.

Recent studies have shown that local inflammation is also related to immunosuppression, and the increase of inflammatory cells in the tumor microenvironment is related to the resistance of ICIs (41, 42). In addition, inflammatory response causes changes in peripheral blood white blood cells, which can be captured by neutrophils and lymphocytes, resulting in increased inflammation burden, which is also associated with poor long-term survival of cancer patients after ICIs (43, 44). Further, single-cell RNA sequencing revealed that neutrophils participate in suppressing the immune microenvironment through the CD80/CD86-CTLA4 signaling axis. *In vitro* experiments also demonstrated that knocking down CD80 and CD86 can reduce the inhibitory function of neutrophils on T cells. Therefore, removing immunosuppressive tumor-related bone marrow cells may be an effective treatment method to promote the efficacy of immunotherapy (45).

However, this study also has some limitations. First, this is a retrospective study, and it is difficult to avoid bias in treatment and nursing. Second, although this study is a multicenter study, since neoadjuvant immunotherapy in gastric cancer is still in the exploratory stage, there are a small number of cases and poor statistical performance. The results need to be further verified by a larger sample size and higher quality research. Thirdly, although we used propensity matching to minimize the bias between clinical baseline data, laboratory results may still be biased due to differences in experimental techniques or reference standards in different centers. In addition, the results of IBI may be interfered by factors other than cancer (such as infection, chronic diseases, etc.), so the interpretation of the results should be carefully screened. Despite these limitations, our study is the first to demonstrate that IBI is a new and simple prognostic factor for immunotherapy in gastric cancer and can be used as a part of risk stratification and follow-up of neoadjuvant therapy to tailor personalized treatment for gastric cancer patients.

## Conclusion

Our study shows that IBI is the best inflammatory factor to predict the prognosis of neoadjuvant immunotherapy for advanced gastric cancer. This study will help to classify patients at risk to guide the next treatment. This study needs a larger sample of prospective studies for further verification.

## Data Availability

The raw data supporting the conclusions of this article will be made available by the authors, without undue reservation.
