# Supplementary material for: Inflammatory burden index as a prognostic marker in patients with advanced gastric cancer treated with neoadjuvant chemotherapy and immunotherapy
Source: Front Immunol. 2025 Jan 21;15:1471399. doi: 10.3389/fimmu.2024.1471399 (PMC11790653; doi:10.3389/fimmu.2024.1471399)

**Supplemental Content** eTables and eFigures

**eTable.1 Seven inflammatory markers in this study**

**eTable.2 Comparison of baseline data between two groups before propensity matching**

**eTable.3 Comparison of baseline data between two groups after propensity matching**

**eFigure1 Time-dependent ROC curves of four inflammatory markers on overall survival**

**eFigure2 X-tile software takes IBI cutoff value**

**eTable.1 Seven inflammatory markers in this study**

| **Inflammation index** | **Inflammation index formulas** |
| --- | --- |
| C-reactive protein（CRP） |  |
| Neutrophil-platelet ratio (NPR) | Neutrophil (uL)/platelet score (uL) |
| Platelet-to-lymphocyte ratio (PLR) | Platelet (uL)/lymphocyte (uL) |
| Systemic-immune-inflammation index(Sll) | Platelet (uL)*neutrophil (uL)/lymphocyte (uL) |
| Modified Glasgow Prognostic Score (mGPs) | C-reactive protein s 10 mg/L and albumin >35 g/L: 0 score  C-reactive protein <10 mg/L or albumin < 35 g/L: 1 score  C-reactive protein>10 mg/L and albumin < 35 g/L: 2 score  C-reactive proteins 10 mg/L and albumin > 35 g/L: 0 score  C-reactive proteins 10 mg/L and albumin < 35 g/L: 0 score  C-reactive protein >10 mg/L: 1 score  C-reactive protein >10 mg/L and albumin < 35 g/L: 2 score |
| Neutrophil-to-lymphocyte ratio (NLR) | Neutrophil(uL)/lymphocyte (/uL) |
| Inflammatory burden index(lBl) | C-reactive protein (mg/L)*neutrophil (uL)/lymphocyte (/uL） |

**eTable.2** Comparison of baseline data between two groups before propensity matching

| **Characteristic** | **High-IBI**  **n=56** | **Low-IBI**  **n=107** | **P.**  **Value** |
| --- | --- | --- | --- |
| Gender, (%) |  |  | 0.420 |
| Female | 16 (28.6%) | 30(28.0%) |  |
| Male | 40 (71.4%) | 77(72.0%) |  |
| Age, year | 65.0 (54.0, 67.0) | 62(56.0,66.0) | 0.224 |
| BMI, kg/m2 | 24.2(±1.8) | 25.2(±2.5) | 0.435 |
| PD1 cycle | 3 (3, 4) | 4 (3, 4) | 0.235 |
| ypT, (%) |  |  | 0.154 |
| 1 | 5 (8.9%) | 16(15.0%) |  |
| 2 | 10(17.9%) | 16 (15.0%) |  |
| 3 | 31(55.3%) | 52(48.6%) |  |
| 4 | 10 (17.9%) | 23 (21.4%) |  |
| ypN, (%) |  |  | 0.07 |
| 0 | 12(21.4%) | 15(14.0%) |  |
| 1 | 19 (33.9%) | 38 (36.0%) |  |
| 2 | 12 (21.4%) | 18(16.4%) |  |
| 3 | 13(23.3%) | 36 (33.6%) |  |
| TRG, (%) |  |  | **0.001** |
| 0 | 3(5.4%) | 14 (13.1%) |  |
| 1 | 8 (14.3%) | 31(29.0%) |  |
| 2 | 9 (16.0%) | 30 (28.0%) |  |
| 3 | 36(64.3%) | 32 (29.9%) |  |
| Surgery method, (%) |  |  | **0.02** |
| Distal gastrectomy | 30 (53.6%) | 32 (30.0%) |  |
| Total gastrectomy | 26(46.4%) | 75 (70. 0%) |  |
| Postoperative complication, No. (%) |  |  | **0.001** |
| Yes | 18（32.1%） | 11（10.3%） |  |
| No | 38（67.9%） | 96（89.7%） |  |
| Treatment-related adverse events, No. (%) |  |  | 0.205 |
| Yes | 33（58.9%） | 68（64.0%） |  |
| No | 23（41.1%） | 39（36.0%） |  |
| CRP | 23.9 (8.4, 37.2) | 4.8 (2.2, 8.6) | **0.034** |
| IBI | 75.2 (63.5, 83.2) | 4.5 (3.9, 19.6) | **0.001** |
| mGPS, (%) |  |  | 0.223 |
| 0 | 35 (62.5%) | 84 (78.5%) |  |
| 1 | 10 (17.9%) | 9 (8.4%) |  |
| 2 | 11 (19.6%) | 14 (13.1%) |  |
| NLR | 3.2 (2.3, 5.7) | 2.9 (1.4, 4.3) | 0.767 |
| NPR*100 | 2.0 (1.4, 3.0) | 1.2 (1.2, 2.3) | 0.334 |
| SII | 319 (195,821) | 292 (240, 917) | 0.398 |
| PLR | 101.8 (79.0, 192.8) | 85.4 (84.5, 194.1) | 0.286 |

**eTable.3** Comparison of baseline data between two groups after propensity matching

| **Characteristic** | **High-IBI**  **n=56** | **Low-IBI**  **n=56** | **P.**  **Value** |
| --- | --- | --- | --- |
| Gender, (%) |  |  | 0.332 |
| Female | 16 (28.6%) | 19(34.0%) |  |
| Male | 40 (71.4%) | 37(66.0%) |  |
| Age, year | 65.0 (54.0, 67.0) | 63(55.0,68.0) | 0.443 |
| BMI, Kg/m2 | 24.2(±1.8) | 23.2(±1.9) | 0.749 |
| PD1 cycle | 3 (3, 4) | 3 (3, 4) | 0.854 |
| ypT, (%) |  |  | 0.271 |
| 1 | 5 (8.9%) | 7(12.5%) |  |
| 2 | 10(17.9%) | 8 (14.3%) |  |
| 3 | 31(55.3%) | 27(48.2%) |  |
| 4 | 10 (17.9%) | 14 (25.0%) |  |
| ypN, (%) |  |  | 0.481 |
| 0 | 12(21.4%) | 11(19.6%) |  |
| 1 | 19 (33.9%) | 20 (35.7%) |  |
| 2 | 12 (21.4%) | 14(25.0%) |  |
| 3 | 13(23.3%) | 11 (19.6%) |  |
| TRG, (%) |  |  | **0.001** |
| 0 | 3(5.4%) | 10 (17.9%) |  |
| 1 | 8 (14.3%) | 15(26.8%) |  |
| 2 | 9 (16.0%) | 11 (19.6%) |  |
| 3 | 36(64.3%) | 20 (35.7%) |  |
| Surgery method, (%) |  |  | 0.541 |
| Distal gastrectomy | 30 (53.6%) | 27(48.2%) |  |
| Total gastrectomy | 26(46.4%) | 29(51.9%) |  |
| Postoperative complication, No. (%) |  |  | **0.001** |
| Yes | 18(32.1%) | 8(14.3%) |  |
| No | 38(67.9%) | 48(85.7%) |  |
| Treatment-related adverse events, No. (%) |  |  | 0.315 |
| Yes | 33(58.9%) | 30(53.6%) |  |
| No | 23(41.1%) | 26(46.4%) |  |
| CRP | 23.9 (8.4, 37.2) | 5.9 (2.5, 9.5) | **0.018** |
| IBI | 75.2 (63.5, 83.2) | 8.8 (6.5, 20.5) | **0.001** |
| mGPS, (%) |  |  | 0.326 |
| 0 | 35 (62.5%) | 38 (67.9%) |  |
| 1 | 10 (17.9%) | 6 (10.7%) |  |
| 2 | 11 (19.6%) | 12 (21.4%) |  |
| NLR | 3.2 (2.3, 5.7) | 2.8 (1.8, 5.3) | 0.564 |
| NPR*100 | 2.0 (1.4, 3.0) | 1.4 (1.3, 2.5) | 0.214 |
| SII | 319 (195,821) | 281 (230, 834) | 0.265 |
| PLR | 101.8 (79.0, 192.8) | 93.2 (80.4, 181.0) | 0.286 |

**eFigure.1** Time-dependent ROC curves of four inflammatory markers on overall survival


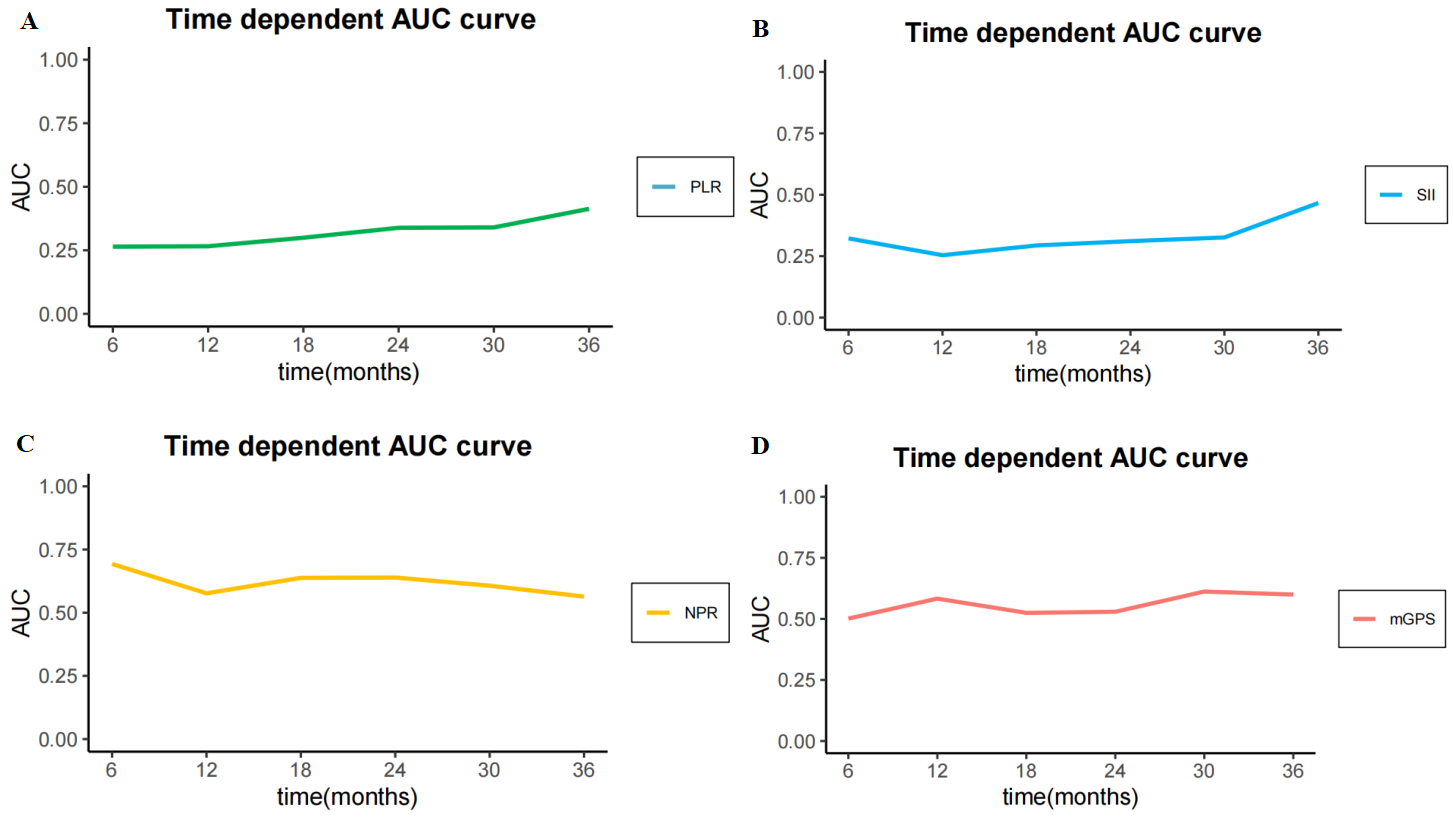


**eFigure.2** X-tile software takes IBI cutoff value


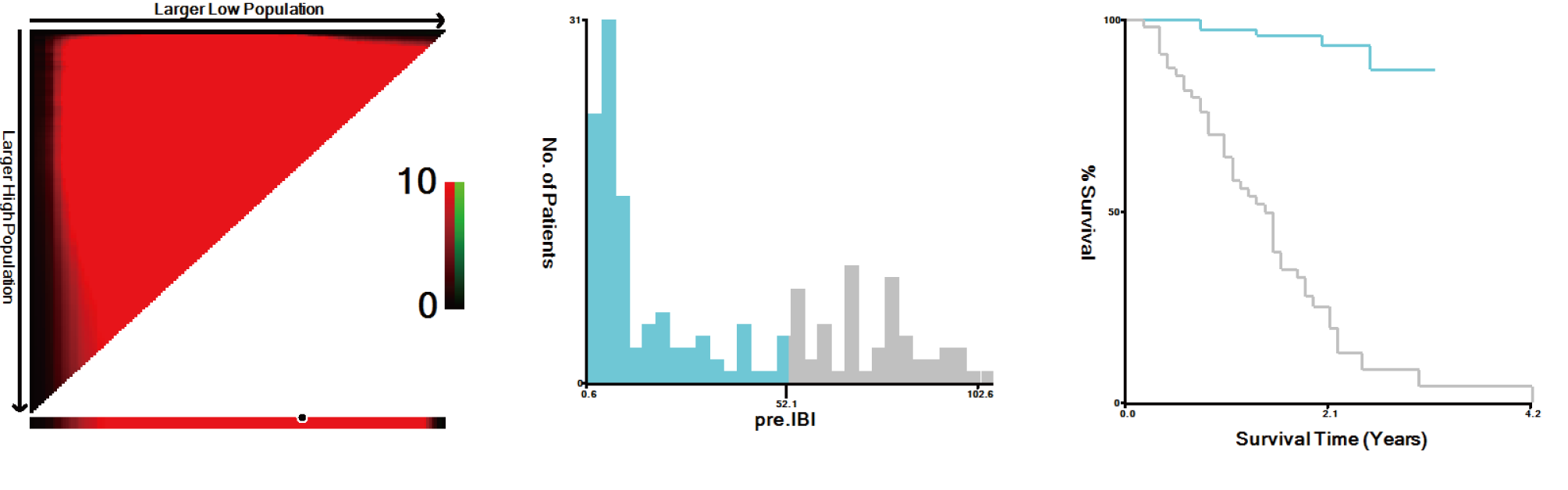

Supplement: Supplementary file 1 [file Table1.docx]
